# Associations with physical activity, sedentary behavior, and premenstrual syndrome among Chinese female college students

**DOI:** 10.1186/s12905-023-02262-x

**Published:** 2023-04-11

**Authors:** Yuqing Shi, Mengyao Shi, Chang Liu, Lu Sui, Ying Zhao, Xiang Fan

**Affiliations:** 1 Department of Physical Education, Shanghai Jiao Tong University, Shanghai, 200240 China; 2 Department of Physical Education, The High school Affiliated to, Southern University of Science and Technology, Shengzhen, 518109 China

**Keywords:** Physical activity, Sedentary behavior, Premenstrual Syndrome, Female students

## Abstract

**Purpose:**

Premenstrual syndrome refers to a set of distressing symptoms experienced before the menstrual flow, which can affect female students’ behavior, cognitive abilities, mental health status, and academic performance. Identifying modifiable risk factors is essential to reduce the prevalence college students’ premenstrual syndrome. We examined associations between premenstrual syndrome and physical activity and sedentary behavior in Chinese female college students.

**Methods:**

In this cross-sectional study, 315 female college students volunteered to participate at a university in Shanghai, China. We measured physical activity and sedentary behavior using the ActiGraph GT3X-BT and assessed premenstrual syndrome using the Premenstrual Symptoms Screening Tool. The data were statistically analyzed using SPSS 24.0 software, and the primary analysis methods included Kruskal-Wallis test and logistic regression analysis.

**Results:**

Among the 221 female college students who met the inclusion criteria, 148 (67.0%) had PMS while 73 (33.3%) did not. After controlling for confounding variables, moderate physical activity and moderate to vigorous intensity physical activity were significantly associated with premenstrual syndrome. There was no correlation between light-intensity physical activity, sedentary behavior, and premenstrual syndrome in the study.

**Conclusion:**

Premenstrual syndrome is prevalent among Chinese female college students. Moderate physical activity and moderate-to-vigorous physical activity can be effective in reducing PMS symptoms.

## Introduction

Premenstrual Syndrome (PMS) refers to a group of physical, emotional, and behavioral symptoms including changes in appetite, irritability, depression, anxiety, lack of concentration, and headaches that some women experience periodically during the late luteal phase (7–14 days before menstruation). These symptoms peak within a week before the start of menstruation and improve or disappear after the period begins [1]. Premenstrual Dysphoric Disorder (PMDD) is a severe form of PMS characterized by symptoms of emotional distress [2]. The American Psychiatric Association lists PMDD as a new diagnostic category for depression in the Diagnostic and Statistical Manual of Mental Disorders Fifth edition (DSM-5) [3]. Women with PMS are more likely to suffer from disorders such as generalized anxiety disorder and bipolar disorder [4–6]. A meta-analysis found that women with PMDD have a fourfold increased risk of suicidal ideation and a sevenfold increased risk of suicide attempts [7]. A strong association between PMS and psychiatric disorders that can negatively impact women’s health and quality of life has been reported [4, 8].

College students are in the process of transitioning from adolescence to adulthood. During this period, their outlook on life is developing and this is also a time when mental illness is more likely to occur [9]. In a meta-analysis conducted in Asia, 59% of high school students and 50.3% of college students reported having PMS [10]. PMS symptoms can lead to absenteeism, decreased academic performance, and non-participation in physical activity, and cause considerable social distress among students [11] .

It is critical to identify factors affecting PMS in order to develop strategies to prevent the occurrence of symptoms. Various demographic, physiological, and sociological factors can contribute to PMS. Age, education level [12], and socioeconomic status [13] may influence PMS incidence. Unstable and challenging economic, political and social environments can negatively affect women’s physical and mental health, with worsening health during the menstrual cycle [14]. Physiological factors such as menstrual-related characteristics and obesity [15] are associated with PMS. Earlier menarche (<12 years), Painful periods, heavy bleeding, and bloating may increase the risk of PMS [16]. PMS is significantly associated with.

A healthy lifestyle is often considered essential for a person’s wellbeing. PMS is closely related to lifestyle with smoking, excessive drinking, and unhealthful eating habits exacerbating PMS symptoms [17]. Physical activity (PA) plays a role in disease prevention and the use PA to promote physical and mental health among women has become a popular social trend [18]. A 2020 systematic review reported that regular exercise appeared to be effective in alleviating PMS somatic and psychological symptoms [19]. Sedentary behavior (SB) is linked with specific physical health indicators in adults, including mortality, cardiovascular disease, type II diabetes, cancer, and obesity [20]. Evidence indicates that sedentary people are more likely to suffer from PMS than active people [21].

Based on the ease of access to screen-based entertainment, modern lifestyles, and improved transportation, PA decreases among young people in late adolescence, while SB increases. These changes are also common among students, particularly those attending university [22]. An anonymous web-based survey found that approximately 52.3% of Chinese college students are physically inactive, particularly with respect to high-intensity PA and specific types of PA such as resistance training, and stretching [23]. Reports indicate that college students are highly sedentary, with SB levels equal to or even exceeding desk-based work [24]. However, sedentary time can lead to health risks even when PA guidelines are followed. A meta-analysis reported that PA could not eliminate the increased risk of death associated with prolonged TV viewing [25].

To date, there is limited and conflicting evidence on relationship between PA, SB, and PMS. Several studies have suggested that regular exercise can relieve premenstrual discomfort and reduce PMS incidence [26, 27], although others have found no association between exercise and PMS [28]. Interestingly, women who exercise more were found to have higher levels of PMS than those who exercise infrequently or never [29].

This study examined the correlation between PA, SB, and PMS in female Chinese college students, taking into account confounding factors.

## Materials and methods

### Participants and procedures

The participants were recruited voluntarily through electronic posters and health promotion lectures at a college in Shanghai, China. To identify eligible participants, researchers conducted interviews with students interested in participating in the study. Those who volunteered to participate were included in the sample. The following inclusion criteria applied: [1] female college students aged 18–30 years old; [2] a regular menstrual cycle (defined as a menstrual cycle of 21–35 days ± 7 days). Exclusion criteria were: [1] use of drugs affecting neurotransmitter secretion, such as oral contra captive, antidepressants, and other psychotropic drugs, in the preceding three months; [2] presence of gynecological or psychiatric disorders; [3] current pregnancy or childbirth. The data were collected and analyzed anonymously.

The study was conducted between October 2021 and March 2022 in the laboratories of the participating college. At the beginning of the experiment, an investigator informed the participants about the study inclusion criteria, its content, the benefits of participating in the project, and the possible inconveniences. Next, the investigator explained the PMS related terms and explained how to conduct the questionnaire. The content of the questionnaire was presented through the software “Questionnaire Star”. The informed consent form, purpose and methods of the survey were explained on the first page, and only respondents who agreed to the survey could move on to the following page. Participants who began to complete the questionnaire were considered to have understood the content of the study and agreed to participate voluntarily. Participants were informed that a person should not answer more than once, and they could withdraw from the study at any time. The students were allowed sufficient time to complete the survey and all participants completed the electronic questionnaire in approximately 5–10 min. The questionnaire collected information on demographics, anthropometric characteristics, and PMS. Then, the survey team members introduced the test requirements and contents to the participants and issued the Actilife GT3X-BT and device wear log. Following the standardized protocol for the accelerometer, participants were asked to wear the accelerometer for seven consecutive days, and complete a form relating to information about device wear. During the testing period, the participants need to maintain normal academic activities and not change their daily routines. To ensure the quality of the data, the researchers reminded and encouraged the participants online daily. At the end of the study, the investigator recovered the Actilife GT3X-BT and device wear log.

Some parts of the questionnaire, such as the question “When was your age at menarche” were considered to be culturally sensitive and certain measures were taken by researchers to account for this. First, we promoted women’s health information before the survey to make college students aware of the importance of the study. Second, the survey was conducted by female researchers with experience in investigative research. Furthermore, the personal data and the identity of participants were protected at all times as each participant was assigned an identifying code (ID) which was used for the survey responses. Only the project principal had access to the ID key code, and survey responses were stored separately from identifying information.

Out of 315 female undergraduates who agreed to participate in this study, 94 (29.8%) were excluded due to withdraw their consent to participate (n = 19) or substandard wear of the accelerometer (n = 75). Finally, 221 participants were included in the analysis. We followed up on the reasons for the short duration of the wear. The main reasons included [1] forgetting to wear the device after removing it, and [2] the wearing position at the hip, which made people inconvenient and embarrassed in daily life.

The study protocol followed the recommendations of the Checklist for Reporting Results of Internet E-Surveys [30], was conducted in compliance with the the guidelines of the Declaration of Helsinki and its amendments. All participants provided informed consent. All procedures were approved by the ethics committee of Shanghai Jiao Tong University (Number: H2020043I).

### Measurements

#### Demographic tool

The general demographic characteristics of the participants were assessed by self-administered questionnaires, and included information about age, body mass index, smoking, drinking, screen time, and menstruation-related variables (age at menarche). Body mass index (BMI) reflects the body obesity index and was calculated by dividing the weight (kg) by the square of the height (m). Data on height and weight were self-reported by the students.

#### Sleep quality

Sleep quality was assessed by the Pittsburgh Sleep Quality Index (PSQI) [31]. It assesses sleep quality and disturbances very comprehensively through 19 individual items designed to assess seven components of sleep quality including subjective sleep quality, sleep latency, sleep duration, habitual sleep efficiency, sleep disturbances, use of sleeping medication, and daytime dysfunction. It has been determined that the Chinese version of the PSQI shows good validity and reliability [32]. The PSQI in the present study showed goodinternal consistency (α = 0.72).

#### Premenstrual symptoms

The study used the Premenstrual Symptoms Screening Tool (PSST) developed by Steiner et al. to measure PMS [33]. This is a self-assessment scale based on the DSM-V criteria for the screening of PMS symptoms. The PSST consists of 19 items. Of these, the first 14 items are symptom dimensions and examine the severity of women’s symptoms during PMS. The remaining five items are effect dimensions and examine how (if at all) these symptoms affect normal activities in various functional areas such as relationships, social activities and home responsibilities. The questionnaire was scored on a four-point Likert scale, with 0 for “absent”, 1 for “mild”, 2 for “moderate”, and 3 for “severe”. PMDD was diagnosed if the following criteria were met: [1] at least one of items 1–4 was rated as 3; [2] at least 4 of items 1–14 were rated as 2; [3] at least one of items 15–19 was rated as 3. The assessment of PMS required the following criteria to be satisfied: [1] at least one of items 1, 2, 3, 4 was rated as 2; [2] at least 4 of item 1–14 were rated as 2; [3] at least one of items 15–19 was rated as 2. Based on the severity of PMS, we divided participants into the following three groups: no/mild PMS, moderate PMS, and severe (PMDD). Hou et al. administered the scale to Chinese college students and conducted reliability analyses with good results [34]. The PSST in the present study showed goodinternal consistency (α = 0.93).

#### Physical activity and sedentary behavior

PA and SB were measured with the ActiGraph GT3X-BT triaxle accelerometer (ActiGraph, Pensacola, Florida, USA) that was worn above the right hip using an elastic belt. The device was initialized at a sample rate of 30 Hz to monitor free-living PA and SB. Participants were instructed to wear the accelerometer for at least seven consecutive days (excluding during contact sports and water activities) and complete daily wear logs. Actilife (Version 6.13.4) software was used to export and analyze the accelerometer data. The study uses algorithms in the software to define the wear time of the device and to collect data related to PA and SB, such as low-intensity PA, moderate to high-intensity PA, and relative energy expenditure. Based on a similar study design, we used the Choi algorithm to verify the wear time. A valid day was defined as ten or more hours of wear time; respondents with four or more valid days were included in the analyses. The accelerometer parameters applied in this study were based on previously published studies on PA among female college students.

To ensure that the results of these study can be compared with those of similar studies, this study used the Freedson Adult algorithm to calculate SB and PA engagement times at different intensities. Freedson cutoffs were applied as follows: SB, defined as < 100 counts per minute (cpm); light physical activity (LPA, 100–1951 cpm); moderate physical activity (MPA, 1952–5724 cpm); vigorous physical activity (VPA, 5725–9498 cpm); and moderate to vigorous physical activity (MVPA, > 1951 cpm). Bouts of at least 10 min of MVPA were derived and used for analysis. Sedentary bouts were defined as bout of at least 30 consecutive minutes with recordings of < 100 cpm [35]. Two sedentary bout variables were defined: [1] total duration of sedentary bouts and [2] number of sedentary bouts [36] and these variables were calculated as the average of all valid days’.

### Statistical analysis

Only participants with complete data were included in the analyses. Statistical analysis was performed using SPSS 24.0 and Excel software. Means and SDs were calculated for normally distributed, continuous variables, and medians and IQRs for variables with a non-normal distribution. The Kruskal-Wallis test was used to determine whether there was a significant difference between the medians of the three PMS groups. Logistic regression models were used to analyze the influencing factors of PMS and to calculate the OR values between different subgroups. To assess cross-sectional associations between PA, SB, and PMS, we included Kcals, time spent in SB, LPA, MPA, VPA, and MVPA as exposure variables in separate negative binomial regression models. This model was selected because the PSST outcome variables were highly positively skewed due to over-dispersion. In line with previous studies, we used units of 15 min for MPA and MVPA, 60 min for LPA and SB, to avoid large numbers of minutes and counts producing very small model coefficients that are hard to interpret. We first ran univariate models, before fully adjusted models. The significance level was set as p < 0.05.

## Results

The mean age of study participants was 21.69 years (SD = 2.50). The mean BMI was 21.71 (SD = 2.52) kg/m^2^. Participants spent a mean time of 6.74 (SD = 2.67) hours per day on screens and ate 3.87 (SD = 1.18) breakfasts per week. Alcohol consumption in the survey was 6.8%, and no participants were smokers. In approximately 25.3% of cases, participants reported an age of menarche of 12 years.

The median reported PMS score was 30.00 (25.00–38.00). Participants spent a median of 669.86 (628.86-714.95) minutes per day in SB, 108.57 (85.61-131.09) minutes in LPA, and 37.42 (27.00-48.04) minutes in MVPA. Table 1 shows the characteristics of the study participants.

**Table 1:** Descriptive characteristics of participants

Various		Participants (n = 221)
Age, years		21.69 (2.50)
Height, cm		163.60 (5.35)
Weight, kg		55.79 (9.15)
Body mass index, kg/m^2^		20.85 (3.43)
Screen time, h per day		6.74 (2.67)
Breakfast, times per week		3.87 (1.18)
Sleep quality		6.52 (2.71)
Smoking		
	Yes	0 (0)
	No	221 (100)
Drinking		
	Yes	15 (6.8)
	No	206 (93.2)
Age of menarche		
	<12 years	56 (25.3)
	≥ 12 years	165 (74.7)
Total PMS score (PSST)		30.00 (25.00–38.00)
PMS symptom dimension		23.00(19.00–29.00)
PMS impact dimension		7.00 (6.00–9.00)
Accelerometer wear time, min per day		824.91 (72.53)
Physical activity, min per day		
	LPA	108.57 (85.61-131.09)
	MPA	35.88 (26.55–46.20)
	VPA	0.53 (0.25–1.43)
	MVPA	37.42 (27.00-48.04)
	TPA	148.17 (120.82-174.97)
Average kcals per day		102.82 (72.48-156.59)
Steps per day		6123.83 (4644.00-7679.17)
SB, min per day		669.86 (628.86-714.95)
Total Sedentary bouts		24.00 (15.00–34.00)
Sedentary bouts per day		177.90 (106.05–333.00)
Average length of Sedentary bouts		48.60 (44.60–56.70)
Total Sedentary breaks		18.00 (9.00–28.00)
Sedentary breaks per day		269.50 (172.75–345.80)
Average length of Sedentary breaks		94.00 (60.10-132.20)

Data are n (%), mean (SD), or median (IQR 25–75).

LPA, light activity; MPA, moderate activity; VPA, vigorous activity; MVPA, moderate-to-vigorous activity; TPA, total physical activity; SB, sedentary behavior.

Table 2 demonstrates differences in PA and SB among female college students, according to different PMS groups. The median daily MVPA was 42.67 (29.71–56.14) in the mild/none group, which was higher than in the moderate 35.94 (25.65–45.90) and severe groups 34.87 (29.23–42.18). MPA was also significantly different between PMS groups. In the mild/none group, energy expenditure was 110.35 (84.02-174.38) and median daily step count was 7073.40 (5202.50-8333.33) and these values were significantly higher than those in the moderate and severe groups (P < 0.05). Compared to the mild/none group, the severe group had a higher SB 697.56 (632.24-749.83), but the moderate group had a lower SB 660.13 (622.85–708.10) although these differences were not significant.

**Table 2:** Differences in PA and SB under PMS subgroups

Various	Mild/None(N = 73)	Moderate(N = 116)	Severe(N = 32)	P
	Median (IQR 25–75)			
SB	678.43 (639.44-712.66)	660.13 (622.85–708.10)	697.56 (632.24-749.83)	0.062
LPA	110.86 (82.89–131.00)	110.75 (85.20-135.29)	101.27 (90.83-118.42)	0.626
MPA	40.60 (29.52–54.42)	34.06 (25.25–43.32)	34.48 (28.35–40.02)	<0.01
VPA	0.62 (0.28–3.24)	0.51 (0.25–1.33)	0.47 (0.17–1.08)	0.144
MVPA	42.67 (29.71–56.14)	35.94 (25.65–45.90)	34.87 (29.23–42.18)	<0.01
TPA	155.67 (125.19-185.12)	146.84 (120.82–172.70)	139.52 (115.31–153.70)	0.193
Average kcals per day	110.35 (84.02-174.38)	100.83 (63.45-159.18)	96.03 (60.87-126.48)	<0.05
Steps per day	7073.40 (5202.50-8333.33)	5842.54 (4485.86-7599.68)	5854.30 (4655.71-6943.54)	<0.05
Total Sedentary bouts	26.00 (16.50–38.00)	22.00 (14.00–34.00)	23.50 (16.50-30.75)	0.336
Sedentary bouts per day	182.90 (116.70-386.40)	179.75 (100.00-14.35)	161.25 (115.83-315.83)	0.433
Average length of Sedentary bouts	49.60 (45.05–61.35)	47.90 (44.00-56.30)	50.15 (45.53–58.45)	0.433
Total Sedentary breaks	21.00 (11.50–31.50)	15.50 (8.25–27.75)	18.00 (10.25–25.50)	0.363
Sedentary breaks per day	276.00 (204.10-348.35)	253.00 (157.00-332.80)	276.70 (166.83-373.18)	0.408
Average length of Sedentary Breaks	89.30 (58.65-121.95)	99.50 (62.20-133.65)	102.65 (71.63-143.55)	0.29

Table 3 shows a binary logistic regression analysis with the presence of PMS as the dependent variable and continuous variable and age, BMI, screen time, breakfast times, sleep quality and categorical variable regularity of menstrual cycle, alcohol consumption as independent variables. The analysis did not include smoking because there were no smokers among the participants. The results showed that longer screen time (OR = 1.18, 95% CI = 1.04, 1.34) and worse sleep quality (OR = 1.35, 95% CI = 1.18, 1.54) were risk factors for PMS.

**Table 3:** Logistic regression analysis on the influencing factors of PMS

Various	β	OR (95%CI)	P
Age	0.05	1.05(0.93,1.19)	0.416
BMI	-0.04	0.96(0.89,1.05)	0.393
Screen time	0.17	1.18(1.04,1.34)	<0.05
Breakfast times	0.01	1.01(0.76,1.33)	0.971
Sleep quality	0.30	1.35(1.18,1.54)	<0. 01
Drinking (yes/no)	-0.46	0.63(0.16,2.49)	0.512
Age of menarche(<12/≥12)	-0.37	0.70(0.34,1.44)	0.326

In Table 4, PMS scores were used as independent variables, while average Kcals and time spend in LPA, MPA, VPA, MVPA, SB were used as dependent variables. In the fully adjusted analysis (Table 4), higher MPA, MVPA and Kcals were associated with a lower PMS score. Each 15 min/day increase in MPA was associated with a 42% decrease in the PMS score. For every 15 min increase of MVPA, PMS scores were 39.0% lower. For all exposures, on the basis of p values, the strength of association with the primary outcome was strongest with MPA and MVPA.

**Table 4:** Associations of PA, SB with PMS

Various	Unadjusted model		Fully adjusted model*	
	OR(95%CI)	p value	OR(95%CI)	p value
LPA (per 60 min)	1.152(0.724, 1.833)	0.550	1.362(0.790, 2.350)	0.266
MPA(per 15 min)	0.589(0.441,0.786)	<0.01	0.580(0.421,0.799)	<0.01
VPA (per min)	0.933(0.847, 1.028)	0.163	0.953(0.861, 1.005)	0.358
MVPA(per 15 min)	0.609(0.465, 0.798)	<0.01	0.610(0.454,0.820)	<0.01
SB (per 60 min)	0.883(0.700,1.114)	0.294	1.114(0.717, 1.731)	0.631
Kcals	0.995(0.991, 0.999)	<0.05	0.996(0.992, 1.000)	0.072

* adjusted for screen time, sleep quality, and accelerometer wear time per day.

## Discussion

This study shows that MPA and MVPA was inversely associated with the PMS score. To the best of our knowledge, this is the first study analyzing associations of objectively measured PA, SB, and PMS after adjusting for confounding variables.

The study included 148 participants with PMS, of whom 52.5% had moderate symptoms and 14.5% had severe symptoms. Previous studies have shown that there is considerable geographical variation in PMS prevalence. A meta-analysis showed that the global prevalence of PMS was 47.8%, with higher rates in Asian countries such as Iran (98%) and Turkey (79%) and lower rates in European countries such as Switzerland (10%) and France (12%) [37]. In addition, socioeconomic conditions, living region, genetic mutations, and family history appears to be associated with PMS [38]. Lifestyle factors such as poor diet, smoking, inactive PA, SB, and overweight/obesity can increase the risk of PMS [17]. A recent study using the Global School-based student Health Survey data reported that 1 in 3 adolescents had lifestyle related risk factors [39], and the two most prominent lifestyle risk factors for non-communicable diseases were low fruit-vegetable intake and physical inactivity [39]. A review showed that globally, in 2016, more than a quarter of all people was not getting sufficient PA, and that PA was lower among women than among men [40].

Participant median (IQR 25–75) time spent in MPA and MVPA was 35.88 (26.55–46.20) and 37.42 (27.00-48.04) minutes per day, respectively, in our study. The World Health Organization has recommended that adults aged 18–64 years old should engage in at least 150 min of MVPA per week, and that the activity should be performed in bouts of at least 10 minutes’ duration. Notably, most participants in our study met the recommended MVPA, with 39.69 min per day recorded. This finding may be explained by the fact that Chinese colleges offer physical education classes to increase MVPA among students.

We found that an additional 15 min of MPA was a significant protective factor for PMS and may result in a 42% decrease in the PMS score. Priya et al. studied the effect on PMS of aerobic exercise at different intensities and similarly concluded that MPA may be a potential prophylaxis for PMS [41]. A meta-analysis showed that PA may alleviate specific psychological, physical, and behavioral symptoms associated with PMS, and assist with management of the global symptom profile [27]. PA may affect PMS symptoms through a variety of psychosocial and biological mechanisms, such as increasing circulating levels of endorphins, positively affecting hypothalamic pituitary gonadal hormone levels in the circulation, improving muscle oxygenation, and regulating mental and emotional states [26]. International guidelines state that health benefits are only achieved when a minimal level of MVPA is achieved [42]. Increasing MVPA has the greatest potential for increasing health benefits for most students by increasing energy expenditure, contributing to obesity prevention and muscular and bone development, reducing anxiety and stress, improving self-esteem, mood, and concentration, and lowering the risk of chronic diseases including CVD, type 2 diabetes, and certain cancers [43–45]. PA may affect depressive symptoms through a variety of psychosocial and biological mechanisms, such as stimulating neuroplasticity in brain regions implicated in depression [46]. Most studies have demonstrated these effects with MPA and MVPA, although LPA and VPA act through similar pathways.

VPA is associated with a greater decrease in the risk of major chronic diseases than MPA [47]. However, previous studies on the correlation between VPA and PMS found negative effects [48]. Mariola et al. compared the prevalence of PMS between female artistic gymnasts and non-athletes and found that more athletes had PMS than controls [49]. Artistic gymnasts may be more prone to menstrual-related disorders due to their low body weight, irregular eating habits and intense training. Furthermore, professional athletes suffer greater levels of mental stress and undergo increased loads of high-intensity training, at risk of metabolic disorders, and other risk factors that may affect PMS. In our study, no correlation was found between VPA and PMS, after controlling for variables.

Previous studies indicated that LPA had a positive effect on physical health outcomes such as cardiometabolic health and mortality [50]. LPA results in some risk reduction relative to inactivity and PA of any intensity should be encouraged in all inactive or insufficiently active persons. However, greater levels of exercise achieve a greater reduction in risk. We consider that the beneficial effects of LPA may be mediated by the activation of duration-specific pathways. However, few studies have examined the effect of LPA on PMS and out study did not find an association between LPA and PMS. Our study recommends greater MPA and MVPA to alleviate PMS. In addition to the intensity of PA, we also examined participant energy consumption and found that Kcals were linked with PMS. Kawabe et al. suggested that a total PA of ≥ 3000 metabolic equivalent minutes/week was associated with milder PMS symptoms irrespective of PA intensity [51].

In a number of studies in diverse populations, including young adults, researchers have identified an independent relationship between SB and acute and chronic conditions, with increased mortality rates and increased risk of cardiovascular diseases, diabetes mellitus, metabolic, and cancer [52]. Our results indicated that participants spent most of their awake time in SB (669.86 min/day). A systematic review involving young Chinese adults recently reported similar findings with over 6 h of SB per day [53]. An international study including 10 countries examined accelerometer-measured SB among adults and found that the average time spent in SB was approximately nine hours [54].

Jalali-Farahani et al. reported that prolonged SB is an important health factor among Tehran students [55]. Bianco et al. found that SB was a risk factor for PMS with a significantly higher prevalence of PMS in the sedentary group (54.1%) than in the exercise group (36.8%) [56]. A further study conducted in 2019 found that 42.5% of women in the exercise group felt pain during their menstrual cycle, compared to 60% in the sedentary group [57]. However, no significant differences were found between SB and PMS in our study. Possible influential factors include the characteristics of the population, and the way sedentary behavior was mensurated and expressed.

Chinese college students spend considerable time in sedentary activities such as sitting in class or reading in the library. Therefore, their SB exhibited more consistency. Although we did not find a correlation between SB and PMS in this study, based on previous research we consider that SB is an important factor affecting PMS.

The major strength of this study is that it examined the relationships between PA, SB, and PMS among Chinese female college students and considered PA as well as PA intensity. Furthermore, this study used accelerometers to assess PA and SB and this approach could be used in future studies to develop more effective preventative strategies for PMS amongst young women. Several limitations should be noted. Our study used questionnaires to assess PMS because of their low cost and ease of administration. However, inherent limitations, such as participant recall bias and an inability to accurately retrospectively recall relevant PMS details, may lead to overestimation or underestimation of PMS. Future research may use daily records to examine PMS. Furthermore, we did not consider differences in physical activity across physiological cycles to PMS, which should be included in future studies for analysis. And the target population was limited to Chinese college students and generalization of these findings to other populations should be considered with caution.

## Conclusion

Premenstrual syndrome is prevalent among Chinese female college students. Moderate physical activity and moderate-to-vigorous physical activity can be effective in reducing PMS symptoms. Given the importance of health behaviors for population health and wellbeing, future prospective research should investigate the causality of these associations and examine the interrelationships between SB types, the level and types of PA, and PMS among young women. The results of this study can be used to inform future interventions to promote well-being among young women. Future public health strategies for the prevention of PMS should target PA and, specifically, promote MPA and MVPA.

## Data Availability

The datasets generated and/or analyzed during the current study are not publicly available due to privacy concerns of participants but are available from the corresponding author on reasonable request.

## Abbreviations

PMS: Premenstrual Syndrome
PMDD: Premenstrual Dysphoric Disorder
DSM-5: Diagnostic and Statistical Manual of Mental Disorders Fifth edition
PA: Physical Activity
SB: Sedentary Behavior
ID: Identifying Code
BMI: Body Mass Index
PSST: Premenstrual Symptoms Screening Tool
CPM: Counts Per Minute
LPA: Light Physical Activity
MPA: Moderate Physical Activity
VPA: Vigorous Physical Activity
MVPA: Moderate to Vigorous Physical Activity
